# Dark Triad traits, alexithymia, and emotion regulation as predictors of depression, anxiety, and stress in clinical and non-clinical samples

**DOI:** 10.3389/fpsyg.2025.1674630

**Published:** 2025-09-23

**Authors:** Leah Hornstein, Daniel Schleicher, Angelika Ecker, Stephanie Kandsperger, Romuald Brunner, Irina Jarvers

**Affiliations:** Department of Child and Adolescent Psychiatry and Psychotherapy, University of Regensburg, Regensburg, Germany

**Keywords:** Dark Triad, personality traits, psychopathology, emotion regulation, alexithymia

## Abstract

**Introduction:**

Emotion regulation and personality traits critically influence mental health. Dark Triad characteristics—Machiavellianism, psychopathy, and narcissism—are linked to distress and maladaptive coping, yet their specific associations with depression, anxiety, and stress remain unclear. This study examined how Dark Triad traits, alexithymia facets, and emotion regulation strategies relate to psychopathological symptoms in a German sample.

**Methods:**

A cross-sectional online survey was completed by 425 adults (72.2% female, 1.4% diverse, *M* = 31.36, *SD* = 12.21 range 18–73 years) recruited from the general population and inpatient and outpatient psychiatric clinics in Regensburg, Germany. Participants completed the Naughty Nine questionnaire as well as the Perth Alexithymia Questionnaire (PAQ), the Emotion Regulation Questionnaire (ERQ), and the Depression Anxiety Stress Scale (DASS-21). Analyses included Kendall’s tau-b correlations, multiple regression with robust standard errors, Kruskal-Wallis tests, and post-hoc comparisons.

**Results:**

Machiavellianism was correlated with depression (*p* = 0.023), anxiety (*p* = 0.018) and perceived stress (*p* < 0.001) and remained an predictor of both anxiety (*β* = 0.13, *p* = 0.013 and perceived stress (*β* = 0.13, *p* = 0.010), even when controlling for alexithymia and emotion regulation. Psychopathy showed positive bivariate associations with depression and expressive suppression but did not predict distress in the multivariate models. Narcissism was modestly correlated with depression, anxiety, and stress (all *p* < 0.05), and emerged as a predictor of perceived stress only (*β* = 0.10, *p* = 0.042). The alexithymia facet difficulty appraising positive feelings (PDAF) contributed additional explained variance in psychopathology beyond Dark Triad traits and emotion regulation (Δ*R*^2^ range: 0.07–0.09, all *p* < 0.01). No significant differences in Dark Triad scores were found across treatment-status groups.

**Discussion:**

Machiavellianism was uniquely associated with higher anxiety and stress, and narcissism with greater perceived stress, psychopathy showed no unique links. Difficulties appraising positive affect (PDAF) accounted for additional variance in all measures of psychopathology. These associations suggest that anxiety-management strategies may suit those high in Machiavellianism, stress-reduction approaches those high in narcissism, and positive-affect training could benefit all by targeting PDAF. Longitudinal studies should confirm these links and evaluate combined trait- and emotion-based interventions.

## 1 Introduction

The Dark Triad of personality describes a grouping of subclinical psychopathy, subclinical narcissism and Machiavellianism, which share a socially aversive, aggressive and emotional coldness (Paulhus and Williams, 2002). In recent years, this construct has increasingly been expanded to include a fourth characteristic, subclinical sadism, resulting in the concept of the Dark Tetrad (Paulhus, 2014). Although these traits are primarily regarded as socially aversive, research shows that they can also negatively affect those who possess them. Individuals who score high on the Dark Triad tend to exhibit higher levels of psychopathological distress and greater use of maladaptive emotion regulation strategies (Wei et al., 2024; Walker et al., 2022; Mojsa-Kaja et al., 2021; Papageorgiou et al., 2023). These individual-level vulnerabilities highlight a broader public health challenge, as mental health issues such as depression, anxiety, and stress remain pervasive societal concerns. A recent study showed that the number of German adults reporting elevated depressive symptoms nearly doubled from 7.5% in 2020 to 14.8% in 2023 (Walther et al., 2025). Globally, suicide was the third leading cause of death among 15- to 29-year-olds in 2021 (World Health Organization, 2025). These numbers underscore the importance of empirical research investigating risk factors and correlates of psychopathological symptoms and manifest mental illnesses.

Psychopathy is marked by impulsivity, low empathy, and emotional coldness and has been positively associated with depressive symptoms and elevated stress levels (Paulhus and Williams, 2002; Wei et al., 2024; Mojsa-Kaja et al., 2021; Bonfá-Araujo et al., 2023; Noser et al., 2014; Papageorgiou et al., 2019). Narcissism involves grandiosity, superiority and dominance, as well as the dependence on external validation (Paulhus and Williams, 2002). Empirical findings regarding the relationship between narcissism and psychopathological symptoms vary considerably, particularly when differentiating between vulnerable and grandiose narcissism. While grandiose narcissism often shows no association with psychopathological symptoms and has even been proposed as a potential protective factor for the individual (Papageorgiou et al., 2023; Bonfá-Araujo et al., 2023; Gómez-Leal et al., 2019), vulnerable narcissism has consistently been linked to anxiety and perceived stress (Birkás et al., 2016; Blasco-Belled et al., 2024). Machiavellianism, characterized by a cynical worldview, manipulative interpersonal behavior and a strategic, cold-blooded approach to social interactions has been associated with various forms of psychological distress, including anxiety sensitivity and heightened stress vulnerability (Papageorgiou et al., 2019; Gómez-Leal et al., 2019; Birkás et al., 2016; Christie and Geis, 1970; Lyons et al., 2019).

While certain personality traits may heighten vulnerability to psychological distress, emotion regulation problems emerge as an additional key factor in the development and maintenance of mental health problems (Gross and John, 2003; Aldao et al., 2010; Barnow, 2012; Sloan et al., 2017). Emotion regulation refers to the process by which individuals influence their own emotional experiences, with different strategies for regulating emotions being associated with distinct mental health outcomes (Gross and John, 2003; Gross, 1998). The Extended Process Model of Emotion Regulation describes a chronological sequence: situation, attention, appraisal, and response, during which various emotion regulation strategies may be implemented (Gross, 1998; Gross, 2015). Among the most extensively studied strategies are cognitive reappraisal and expressive suppression (Gross, 2015). Cognitive reappraisal involves reinterpreting an event or its potential consequences in order to modify the anticipated emotional response in advance, whereas expressive suppression refers to efforts to inhibit or conceal the outward expression of emotions once the emotion has already been elicited (Gross, 2015). Research shows that suppression is positively associated with higher levels of depressive and anxiety symptoms, while the use of cognitive reappraisal tends to be linked to more favorable emotional outcomes (Gross and John, 2003; Dawel et al., 2021).

Beyond specific strategies, another construct closely tied to emotion regulation and mental health is alexithymia, considered a transdiagnostic factor associated with increased vulnerability to psychopathology (Weissman et al., 2020). Alexithymia is characterized by difficulties in identifying (DIF) and describing (DDF) one’s own emotions, as well as an externally oriented thinking style (EOT)—that is, a tendency to focus attention away from internal emotional experiences (Sifneos, 1973; Preece et al., 2017). The Attention-Appraisal Model integrates alexithymia within the framework of the Process Model of Emotion Regulation, suggesting that difficulties in attention and appraisal prevent affected individuals from selecting effective emotion regulation strategies (Preece and Gross, 2023). The relationships between the Dark Triad traits and emotion regulation as well as alexithymia differ across the individual traits. Psychopathy has been linked to a greater use of expressive suppression and a reduced tendency to employ cognitive reappraisal (Walker et al., 2022). In previous studies, psychopathy was positively associated with higher scores on general alexithymia and correlated with all its subdomains (Jonason and Krause, 2013; Burghart and Mier, 2022; Di Tella et al., 2024; Cairncross et al., 2013). Vulnerable narcissism has been associated with increased use of expressive suppression, indicating a tendency toward maladaptive regulation strategies (Walker et al., 2022). Findings on the relationship between narcissism and alexithymia remain mixed. Some studies have reported positive correlations, particularly with the alexithymia dimensions DIF and DDF, whereas others have found negative relationships (Jonason and Krause, 2013; Cairncross et al., 2013). Previous findings concerning emotion regulation in individuals with Machiavellian traits are inconsistent. While a recent meta-analysis found no correlation between Machiavellianism and the use of emotion regulation strategies (Walker et al., 2022), another study showed a positive correlation between Machiavellianism and increased use of expressive suppression (Akram and Stevenson, 2021). Moreover, Machiavellianism has been positively linked to alexithymia in various studies (Jonason and Krause, 2013; Di Tella et al., 2024; Cairncross et al., 2013; Al Aïn et al., 2013).

Taken together, the characteristics of the Dark Triad have been previously linked, to varying degrees, with psychopathological symptoms, patterns of emotion regulation, and alexithymia. Building on these associations, the distinct psychological profiles of the three Dark Triad traits warrant closer examination.

Although a growing body of research has explored associations between the Dark Triad traits and psychological distress, most findings have emerged from studies using non-clinical, often student-based samples in Anglo-American contexts. Research investigating these traits in clinical populations remains scarce, and cross-cultural comparisons suggest that the strength and direction of these associations may vary depending on sociocultural factors (Papageorgiou et al., 2023). Furthermore, few studies have examined how these traits relate simultaneously to both emotion regulation and alexithymia within the same sample. To date, findings have largely been fragmented across separate domains, limiting a more integrated understanding of how dark personality traits contribute to psychopathology. The present research addresses these gaps by examining these relationships in both clinical and non-clinical German-speaking participants.

The aim of the current study was to investigate how Dark Triad personality traits relate to psychopathological symptoms, specifically depression, anxiety, and perceived stress. It also examines the relationship between these traits and the use of cognitive reappraisal and expressive suppression as emotion regulation strategies. Based on prior findings, it is expected that higher levels of psychopathy will be associated with greater depressive symptoms, increased use of expressive suppression, and reduced use of cognitive reappraisal. Machiavellianism is hypothesized to be positively related to anxiety symptoms, elevated stress, and greater use of expressive suppression. Narcissistic traits are also expected to be associated with psychopathological symptoms and emotion regulation strategies, though findings in the literature have been more inconsistent, particularly when differentiating between grandiose and vulnerable narcissism.

To account for sample variability, both clinical and non-clinical participants are included, enabling exploratory comparisons of Dark Triad traits, psychopathological symptoms and emotion regulation across treatment contexts.

Furthermore, the study explores the role of alexithymia, focusing on whether its associations with Dark Triad traits and psychopathological symptoms differ by treatment status and alexithymia dimensions. In addition, the study explores which subdimensions and emotion valences of alexithymia show particularly strong associations with the individual Dark Triad traits. Beyond bivariate associations, we test whether Dark Triad traits and alexithymia explain additional variance in psychological distress above and beyond sociodemographic factors, treatment status, and emotion regulation strategies.

In summary, the study aims not only to examine bivariate associations between personality, emotion regulation, and psychopathology, but also to test whether Dark Triad traits and alexithymia contribute unique variance to mental health outcomes beyond established predictors.

## 2 Materials and methods

### 2.1 Participants and procedure

Participants were recruited from both the general population and the Clinic and Polyclinic for Psychiatry and Psychotherapy as well as the Polyclinic for Child and Adolescent Psychiatry, Psychosomatics, and Psychotherapy of the University of Regensburg at the medbo District Hospital Regensburg. Recruitment took place between January and June 2025 via multiple channels, including flyers and social media. Although adolescents (aged 14–17) were initially included, their number was too small for meaningful subgroup analyses. Therefore, only data from adult participants were used (*N* = 425). Participants in the clinical subsample reported heterogeneous psychiatric diagnoses (multiple responses allowed). The most frequently selected diagnoses were anxiety disorders (28.5%), depressive disorders (28.0%), trauma-related disorders (13.9%), AD(H)D (8.5%), and eating disorders (7.1%). Less frequently reported problems included psychotic disorders, bipolar disorders, obsessive-compulsive problems, somatoform disorders, autism spectrum disorder, and personality disorders. The survey was administered online using PsyToolkit (Stoet, 2010; Stoet, 2017). Participants had the opportunity to win one of 50 vouchers worth €25 after completing the survey. Prior to giving consent all respondents were informed that the survey would include questions addressing topics such as depression, anxiety, stress and self-injurious thoughts and behaviors. Following consent, participants first completed demographic questions and indicated whether they were currently or previously in psychiatric treatment. Various psychometric instruments were then presented in fixed order, interspersed with several attention-checks. Participation was voluntary and could be discontinued at any point by closing the browser window. Contact details for local mental-health services were provided in case of emotional distress. Ethical approval was obtained from the Ethics Committee of the University of Regensburg (reference 22-2,985-104), and the study was preregistered in the German Clinical Trials Register (DRKS00029332). A subsample of this ongoing validation study has been previously published by Baumann et al. (2025), examining the relationship between non-suicidal self-injury and alexithymia.

### 2.2 Questionnaires

#### 2.2.1 Naughty Nine

The Naughty Nine questionnaire was used to assess Dark Triad personality traits. It represents a psychometrically optimized German version of the Dirty Dozen, comprising three items per subscale (Webster and Jonason, 2010; Küfner et al., 2015). The total of nine items is rated on a 9-point Likert scale ranging from 1 (completely disagree) to 9 (completely agree). In the past, the subscales and the overall scale of the Naughty Nine have demonstrated good internal consistency, and stability, as well as a satisfactory relationship to the standard measures of the Dark Triad (Küfner et al., 2015). In the present sample the three subscales demonstrated acceptable to good internal consistency (McDonald’s *ω* = 0.69 for psychopathy, 0.86 for narcissism and 0.79 for Machiavellianism).

#### 2.2.2 Depression anxiety stress scale (DASS-21)

The Depression Anxiety Stress Scale (DASS-21) is a self-report instrument designed to assess symptoms of depression, anxiety, and stress in adults, originally developed for use with pain patients (Lovibond and Lovibond, 1995). In the present study, the German short version was administered. It consists of 21 items rated on a 4-point Likert scale ranging from 1 (did not apply to me at all) to 4 (applied to me very much or most of the time). The German version of the DASS-21 has demonstrated good reliability and validity across various populations, extending beyond its original application in pain patients (Nilges and Essau, 2015). In the present sample McDonald’s omega indicated good reliability for all subscales (*ω* = 0.91 for Depression, 0.84 for Anxiety and 0.88 for Stress).

#### 2.2.3 Emotion regulation questionnaire (ERQ)

To assess emotion regulation strategies, the German version of the Emotion Regulation Questionnaire (ERQ) was used (Abler and Kessler, 2011). The ERQ was developed to measure individual tendencies in the use of expressive suppression (considered a maladaptive emotion regulation strategy) and cognitive reappraisal (considered an adaptive response-focused strategy). It comprises 10 items rated on a 7-point Likert scale ranging from 1 (strongly disagree) to 7 (strongly agree), with higher scores indicating more frequent use of the respective strategy. Previous studies have demonstrated good reliability and validity of the German version (Abler and Kessler, 2011). In this sample both subscales of the ERQ showed good internal consistency (McDonald’s *ω* = 0.80 for suppression, 0.85 for reappraisal).

#### 2.2.4 Perth alexithymia questionnaire (PAQ)

The Perth Alexithymia Questionnaire (PAQ) is based on the Attention–Appraisal Model of alexithymia and consists of 24 items rated on a 7-point Likert scale ranging from 1 (strongly disagree) to 7 (strongly agree) (Preece et al., 2018; Kaemmerer et al., 2021). It distinguishes between five subscales that capture different facets of alexithymia for negative and positive emotions: Negative Difficulty Identifying Feelings (N-DIF), Positive Difficulty Identifying Feelings (P-DIF), Negative Difficulty Describing Feelings (N-DDF), Positive Difficulty Describing Feelings (P-DDF), and Externally Oriented Thinking (EOT). Based on these, theoretically meaningful composite scores can be computed: the General-Externally Oriented Thinking scale (GEOT), the Positive Difficulty Appraising Feelings scale (PDAF; combining P-DIF and P-DDF), and the Negative Difficulty Appraising Feelings scale (NDAF; combining N-DIF and N-DDF) (Kaemmerer et al., 2021). In addition, a total score of alexithymia can be calculated by summing all items, representing an overall value of difficulties in attending to and appraising one’s own emotional experiences, both negative and positive. In the present study, the German version of the PAQ was used and the three subscales GEOT, PDAF, and NDAF were selected to allow for a distinction between emotional valence in appraisal-related difficulties (Kaemmerer et al., 2021). All subscale scores demonstrated good to excellent internal consistency and reliability in the past, with Cronbach’s alpha values ranging from *α* = 0.87 to 0.91 (Preece et al., 2018). The three subscales of the German PAQ used in this study showed very good internal consistency in the present sample (McDonald’s *ω* = 0.97 for the total score, 0.91 for GEOT, 0.92 for NDAF and PDAF).

### 2.3 Statistical analysis

All statistical analyses were carried out using R (version 4.5.0) and RStudio (R Core Team, 2025). Descriptive statistics, including means, standard deviations and ranges where applicable were generated for all major study variables. Internal consistency was assessed using McDonald’s omega coefficients. The full sample (*N* = 425) was subdivided into three groups based on mental health treatment history: currently in treatment, past treatment, and never in treatment. As the data did not meet assumptions of normality, non-parametric procedures were applied for correlation and group comparisons. Kruskal-Wallis tests were used to examine group differences in psychopathological symptoms, emotion regulation and Dark Triad traits. Post-hoc pairwise Wilcoxon comparisons were adjusted using the False Discovery Rate (FDR) (Benjamini and Hochberg, 1995). To test whether the association between alexithymia and Dark Triad traits differed by treatment status, interaction terms were included in the linear models. Bivariate relationships between dark personality traits, psychopathological symptoms, and emotion regulation strategies were assessed using Kendall’s tau-b correlation coefficients. Multiple linear regression analyses were conducted in two steps to examine the predictive value of Dark Triad traits, emotion regulation strategies, and alexithymia for depression, anxiety, and perceived stress. Covariates included age, gender (coded as 1 = female, 2 = male), and mental health treatment status (past and current). Participants reporting a gender other than male or female were excluded from the regression due to small sample size (*n* = 9). A backward selection approach was used to identify the most robust predictors while minimizing the risk of overfitting, given the number of variables examined and the moderate sample size. Model selection followed a backward stepwise procedure based on the Akaike Information Criterion (AIC) (Akaike, 1973). Robust standard errors (HC3) were used to adjust for heteroskedasticity. To confirm the validity of the regression results, we conducted several robustness checks. (a) Bootstrapped regression coefficients (1,000 samples, *n* = 416) yielded confidence intervals highly similar to those reported in the main analyses, indicating stability of estimates. (b) Examination of influence diagnostics (Cook’s distance) indicated that no single case substantially altered the results (all values < 1.0). (c) Variance Inflation Factors (VIFs) were all below 3, suggesting that multicollinearity was not a concern. Together, these analyses support the robustness of the reported findings. All correlation and regression coefficients are reported with exact *p*-values and standardized estimates where appropriate. Effect sizes were interpreted according to Cohen’s guidelines (Cohen, 1988).

## 3 Results

### 3.1 Descriptive results

**Figure 1:**
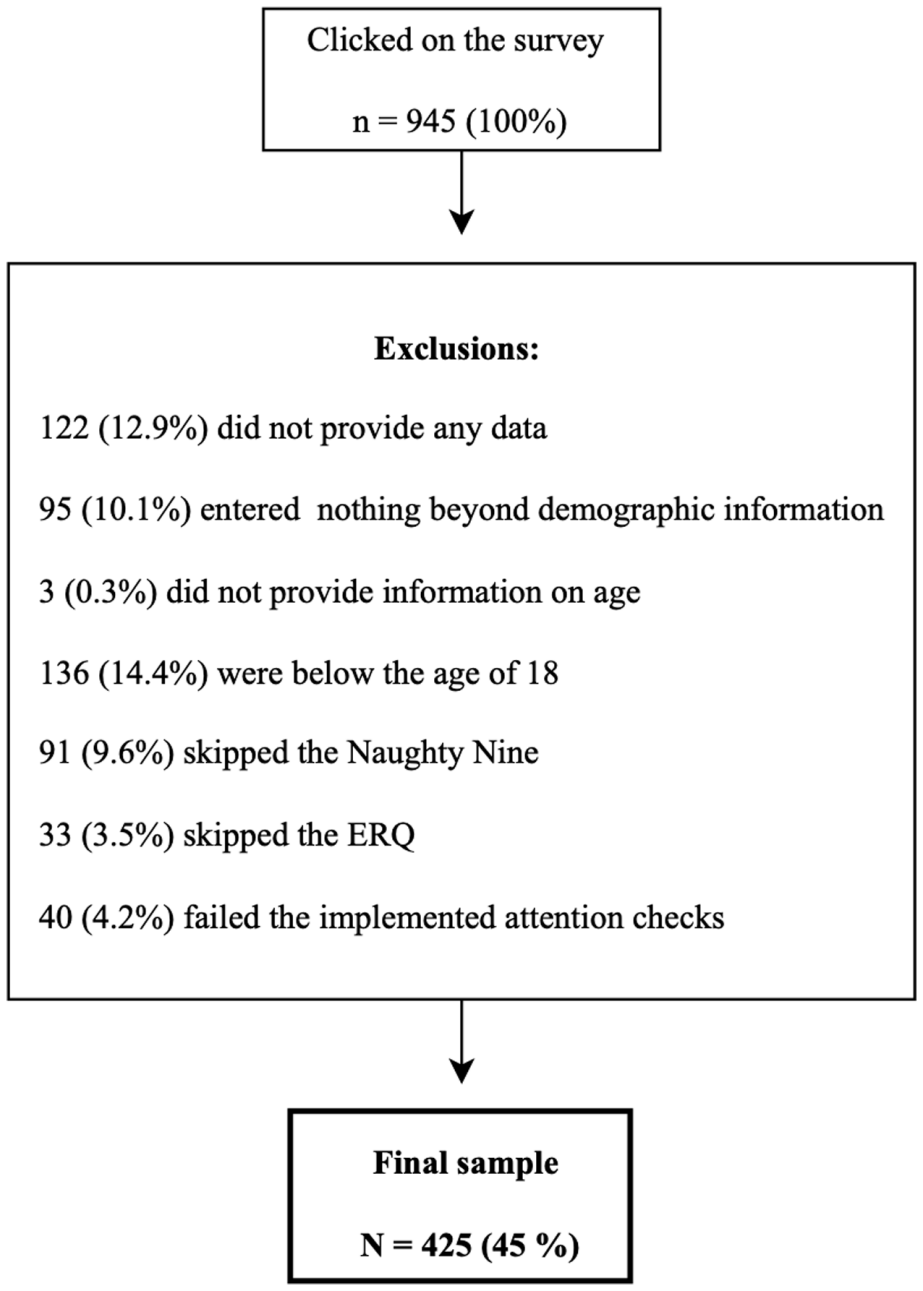
Flow diagram illustrating participant recruitment and exclusion. ERQ, Emotion Regulation Questionnaire.

Of 945 persons who clicked on the survey, 520 were excluded for the following reasons: 122 provided no data, 95 completed only demographics and 3 failed to report age, 91 persons skipped the Naughty Nine, 33 persons skipped the ERQ, 136 were below 18 years of age, and 40 failed the implemented attention checks. The flow of participants through the study, including exclusion criteria, is depicted in Figure 1. The resulting sample consisted of 425 adults (72.2% female, 1.4% diverse, *M* = 31.36 years, *SD* = 12.21 range 18–73 years) and was subdivided in three groups, depending on psychological or psychiatric treatment status: 88 currently in treatment, 99 previously in treatment, and 238 have never been in psychiatric or psychological treatment. The three treatment groups showed broadly similar patterns in terms of age, gender distribution, and educational attainment (see Table 1 for subgroup demographics).

**Table 1:** Descriptive sample characteristics.

Variable	Category	Sample		Current psychiatric or psychological treatment		Psychiatric or psychological treatment in the past		No psychiatric or psychological treatment	
		*N* = 425	%	*n* = 88	%	*n* = 99	%	*n* = 238	%
Gender	Female	307	72.24	66	75.00	77	77.78	164	68.91
	Male	109	25.65	19	21.59	20	20.20	70	29.41
	Diverse	6	1.41	3	3.41	1	1.01	2	0.84
	Other	1	0.24	0	0.00	1	1.01	0	0.00
	No information	2	0.47	0	0.00	0	0.00	2	0.84
Education/degree	Still in school	11	2.59	5	5.68	4	4.04	2	0.84
	No degree	1	0.24	1	1.14	0	0.00	0.00	0.00
	Hauptschule	11	2.59	3	3.41	3	3.03	5	2.10
	Mittlere Reife	79	18.59	19	21.59	18	18.18	42	17.65
	Fachhoch- schulreife	58	13.65	12	13.64	10	10.10	36	15.13
	Abitur	244	57.41	44	50.00	57	57.58	143	60.08
	Other	21	4.94	4	4.55	7	7.07	10	4.20

	Mean (*SD)*	Mean (*SD*)	Mean (*SD*)	Mean (*SD*)
Age (years)	31.36 (12.21)	32.98 (12.91)	31.82 (12.60)	30.57 (11.75)
Naughty Nine Psychopathy	2.70 (1.67)	2.57 (1.62)	2.85 (1.67)	2.69 (1.68)
Naughty Nine Narcissism	4.55 (2.25)	4.59 (2.30)	4.63 (2.20)	4.51 (2.26)
Naughty Nine Machiavellianism	3.04 (1.93)	2.74 (1.70)	3.39 (2.12)	3.01 (1.91)
DASS Depression	5.80 (5.25)	8.73 (5.81)	6.70 (5.51)	4.34 (4.32)
DASS Anxiety	4.33 (4.42)	6.20 (4.61)	5.34 (4.40)	3.31 (4.02)
DASS Stress	7.65 (5.05)	9.85 (5.34)	8.93 (5.43)	6.30 (4.31)
ERQ Suppression	3.60 (1.38)	3.64 (1.39)	3.52 (1.44)	3.63 (1.36)
ERQ Reappraisal	4.41 (1.18)	3.83 (1.17)	4.33 (1.22)	4.66 (1.09)
PAQ Total score	72.23 (31.07)	83.64 (33.50)	71.22 (32.65)	68.43 (28.48)
PAQ GEOT	24.15 (11.74)	26.03 (12.30)	23.54 (12.18)	23.70 (11.32)
PAQ PDAF	21.12 (11.24)	25.99 (12.65)	20.65 (11.63)	19.52 (9.99)
PAQ NDAF	26.96 (12.37)	31.61 (12.99)	27.04 (13.36)	25.21 (11.27)

Hauptschule (school degree after 9 years of elementary school), Mittlere Reife (degree after 10 years of intermediate secondary school), Fachhochschulreife (second-highest school degree, entitlement to study at universities of applied sciences), Abitur (highest school degree, general university entrance qualification), ERQ, Emotion Regulation Questionnaire; PAQ = Perth Alexithymia Questionnaire; PDAF, Positive Difficulty Appraising Feelings; NDAF, Negative Difficulty Appraising Feelings; GEOT, General Externally Oriented Thinking.

### 3.2 Kendall’s tau-b correlations

Bivariate Kendall’s tau-b correlations are presented in Table 2. When examining Dark Triad traits, psychopathy was positively associated with both depressive symptoms (*τ* = 0.10, *p* = 0.003) and expressive suppression (*τ* = 0.14, *p* < 0.001), while no significant association was found with cognitive reappraisal (*τ* = −0.04, *p* = 0.153). Machiavellianism was positively related to anxiety (*τ* = 0.07, *p* = 0.018) and stress levels (*τ* = 0.11, *p* < 0.001). However, no significant association was found with expressive suppression (*τ* = −0.01, *p* = 0.570). Narcissistic traits also showed consistent positive associations with depressive symptoms (*τ* = 0.11, *p* < 0.001), anxiety (*τ* = 0.08, *p* = 0.016), and stress (*τ* = 0.11, *p* < 0.001). However, neither expressive suppression (*τ* = −0.01, *p* = 0.688) nor cognitive reappraisal (*τ* = 0.03, *p* = 0.391) were significantly related to narcissism. With regard to alexithymia, psychopathy showed significant positive associations with the total PAQ score (*τ* = 0.23, *p* < 0.001) and the three PAQ subscales: GEOT (*τ* = 0.23, *p* < 0.001), NDAF (*τ* = 0.19, p < 0.001), and PDAF (*τ* = 0.19, *p* < 0.001). In contrast, narcissism and Machiavellianism showed only weak positive correlations with the NDAF subscale (*τ* = 0.08, *p* = 0.019–0.027) but no significant associations with the PAQ total score or the other subscales (all other *p* > 0.05). The total score of alexithymia was significantly positively associated with depressive symptoms (*τ* = 0.33, *p* < 0.001), anxiety (*τ* = 0.28, *p* < 0.001), and perceived stress (*τ* = 0.25, *p* < 0.001).

**Table 2:** Kendall’s Tau correlations between Dark Triad traits, psychopathological symptoms, emotion regulation, and alexithymia.

Variable	Psychopathy		Narcissism		Machiavellianism	
	*τ*	*p*	*τ*	*p*	*τ*	*p*
DASS depression	0.10	0.003	0.11	<0.001	0.08	0.023
DASS anxiety	0.09	0.015	0.08	0.016	0.07	0.018
DASS stress	0.11	0.002	0.11	<0.001	0.11	<0.001
ERQ suppression	0.14	<0.001	−0.01	0.688	−0.01	0.570
ERQ reappraisal	−0.04	0.153	0.03	0.391	0.00	0.978
PAQ total score	0.23	<0.001	0.05	0.113	0.07	0.052
PAQ PDAF	0.19	<0.001	0.05	0.111	0.05	0.182
PAQ NDAF	0.19	<0.001	0.08	0.019	0.08	0.027
PAQ GEOT	0.23	<0.001	0.00	0.948	0.06	0.080

ERQ, Emotion Regulation Questionnaire; PAQ, Perth Alexithymia Questionnaire; PDAF, Positive Difficulty Appraising Feelings; NDAF, Negative Difficulty Appraising Feelings; GEOT, General Externally Oriented Thinking.

### 3.3 Regression analyses

Multiple linear regression analyses with backward selection were conducted to examine whether Dark Triad traits, emotion regulation strategies, age, and treatment status predict psychopathological symptoms. Table 3 summarizes the detailed results of the multiple regression analyses predicting depression, anxiety, and stress.

**Table 3:** Multiple regression analyses predicting depression, anxiety, and stress.

Dependent variable	Predictors	*B*	*ß*	*SE* (robust)	CI 95%	*p* (robust)	*R* ^2^
Depression	Intercept	3.86	0.00	1.50	0.92–6.81	0.010	0.35
	Gender	−1.44	−0.12	0.57	−2.56 - -0.33	**0.012**	
	Narcissism	0.22	0.10	0.11	0.00–0.44	0.051	
	Machiavellianism	0.26	0.10	0.15	−0.04 - 0.55	0.085	
	ERQ Suppression	0.35	0.09	0.21	−0.07 - 0.77	0.100	
	ERQ Reappraisal	−0.96	−0.22	0.20	−1.35 - -0.56	**<0.001**	
	Current treatment	2.53	0.20	0.65	1.25–3.80	**<0.001**	
	Past treatment	1.59	0.13	0.50	0.60–2.58	**0.002**	
	PAQ PDAF	0.09	0.20	0.04	0.02–0.16	**0.009**	
	PAQ NDAF	0.04	0.10	0.03	−0.02 - 0.10	0.184	
	PAQ GEOT	0.04	0.09	0.04	−0.04 - 0.11	0.305	
Anxiety	Intercept	4.86	−0.00	1.31	2.29–7.43	<0.001	0.31
	Age	−0.06	−0.17	0.02	−0.09 - -0.03	**<0.001**	
	Gender	−1.90	−0.19	0.41	−2.71 - -1.09	**<0.001**	
	Machiavellianism	0.29	0.13	0.12	0.06–0.52	**0.013**	
	ERQ Reappraisal	−0.29	−0.08	0.18	−0.65 - 0.07	0.111	
	Current treatment	1.76	0.16	0.51	0.75–2.77	**<0.001**	
	Past treatment	1.59	0.15	0.44	0.72–2.46	**<0.001**	
	PAQ PDAF	0.11	0.29	0.03	0.06–0.17	**<0.001**	
	PAQ NDAF	0.04	0.10	0.03	−0.01 - 0.09	0.163	
Stress	Intercept	7.80	0.00	1.37	5.11–10.50	<0.001	0.31
	Gender	−2.70	−0.24	0.47	−3.63 - -1.79	**<0.001**	
	Narcissism	0.22	0.10	0.11	0.01–0.43	**0.042**	
	Machiavellianism	0.35	0.13	0.14	0.09–0.62	**0.010**	
	ERQ Reappraisal	−0.66	−0.16	0.20	−1.04 - -0.27	**<0.001**	
	Current treatment	1.89	0.15	0.61	0.69–3.09	**0.002**	
	Past treatment	1.78	0.15	0.51	0.78–2.78	**<0.001**	
	PAQ PDAF	0.16	0.35	0.02	0.12–0.20	**<0.001**	

*n* = 416, Significant predictors are displayed in bold (*p* < 0.05), ERQ, Emotion Regulation Questionnaire; PAQ, Perth Alexithymia Questionnaire.

The final model for depressive symptoms was significant and explained 35% of the variance in depressive symptoms [adjusted *R^2^* = 0.35, *F*(10, 405) = 23.75, *p* < 0.001]. Significant predictors included gender, cognitive reappraisal, current treatment status, past treatment status, and the PDAF dimension of alexithymia. Narcissism and Machiavellianism showed marginal associations with depressive symptoms but were not significant.

The multiple linear regression predicting anxiety symptoms was significant and explained 31% of the variance in anxiety symptoms [adjusted *R^2^* = 0.32, *F*(8, 407) = 24.13, *p* < 0.001]. Significant predictors included age, gender, Machiavellianism, current and past treatment status, and the PDAF dimension of alexithymia. Cognitive reappraisal and the NDAF dimension of alexithymia were not significantly associated with anxiety symptoms.

Similarly, the regression model for perceived stress was significant and explained 31% of the variance [adjusted *R^2^* = 0.32, *F*(7, 408) = 27.08, *p* < 0.001]. Significant predictors were gender, narcissism, Machiavellianism, cognitive reappraisal, current and past treatment status, and the PDAF dimension of alexithymia.

### 3.4 Group comparisons

**Figure 2:**
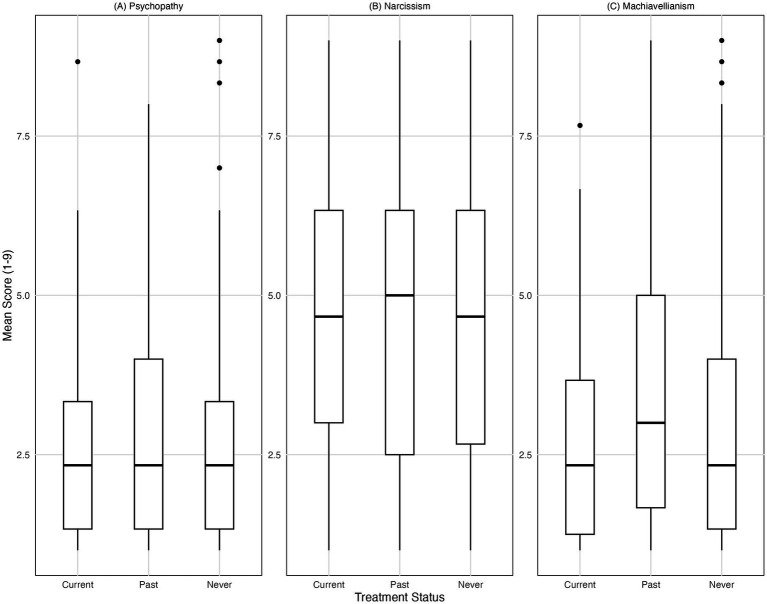
Mean scores for **(A)**, Psychopathy, **(B)** Narcissism, and **(C)** Machiavellianism across treatment status groups (Current, Past, Never). Scores range from 1 to 9. Boxes represent the interquartile range (IQR) from the first (Q1) to the third quartile (Q3); the horizontal line within each box indicates the median. Whiskers extend to the most extreme data points within 1.5 times the IQR from the quartiles. Data points outside this range are shown individually as outliers.

**Figure 3:**
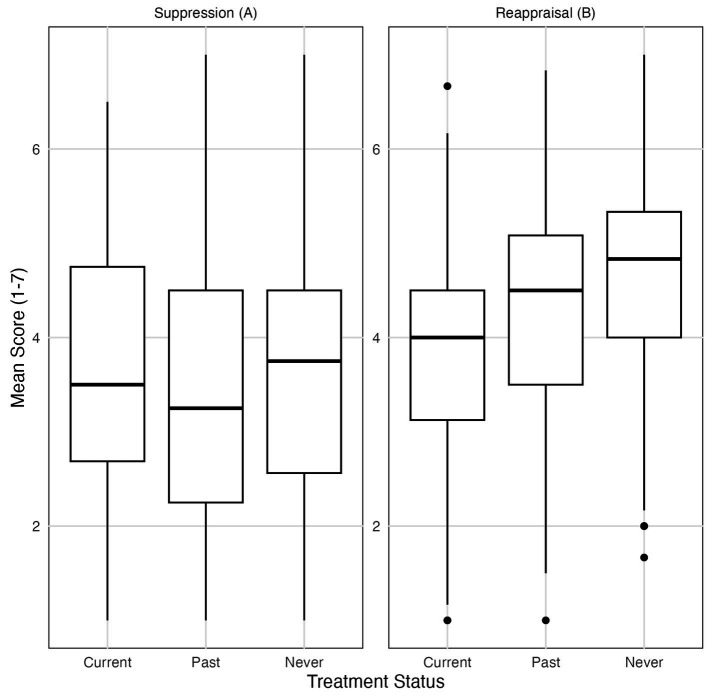
Mean scores for **(A)** Cognitive Reappraisal and **(B)** Expressive Suppression across treatment status groups (Current, Past, Never). Scores range from 1 to 7. Boxes represent the interquartile range (IQR) from the first (Q1) to the third quartile (Q3); the horizontal line within each box indicates the median. Whiskers extend to the most extreme data points within 1.5 times the IQR from the quartiles. Data points outside this range are shown individually as outliers.

No significant differences in Dark Triad traits were found between participants who were currently in treatment, those with past treatment, and those who had never received treatment as shown in Figure 2. Specifically, there were no significant group differences for psychopathy [*χ^2^*(2) = 1.81, *p* = 0.405], narcissism [*χ^2^*(2) = 0.31, *p* = 0.856] or Machiavellianism [*χ^2^*(2) = 4.24, *p* = 0.120]. Kruskal-Wallis tests revealed significant differences in depressive symptoms, anxiety, and perceived stress depending on treatment status. For depression [*χ^2^*(2) = 47.88, *p* < 0.001, *η^2^* = 0.11], for anxiety [*χ^2^*(2) = 49.81, *p* < 0.001, *η^2^* = 0.11] and for stress [*χ^2^*(2) = 37.77, *p* < 0.001, *η^2^* = 0.08]. *Post-hoc* pairwise Wilcoxon tests with FDR correction showed that participants currently in treatment reported significantly higher levels of depressive symptoms, anxiety, and stress than those never treated (all *p* < 0.001). Past treatment was also associated with higher scores compared to never treated (all *p* < 0.001). No significant differences were found between current and past treatment groups for anxiety (*p* = 0.55) or stress (*p* = 0.60), but depressive symptoms differed between these groups (*p* = 0.034). Regarding emotion regulation, significant group differences were found for cognitive reappraisal, *χ^2^*(2) = 32.41, *p* < 0.001, *η^2^* = 0.07 (*f* = 0.28), but not for expressive suppression, *χ^2^*(2) = 0.86, *p* = 0.660. Post-hoc tests indicated that participants currently in treatment reported significantly lower use of cognitive reappraisal than those with past treatment (*p* = 0.009) and those never treated (*p* < 0.001). Participants with past treatment also reported significantly lower use of cognitive reappraisal than those never treated (*p* = 0.023). Mean scores for cognitive reappraisal and expressive suppression are illustrated in Figure 3.

### 3.5 Moderation analysis

To examine whether the association between alexithymia and Dark Triad traits varies by treatment status (clinical vs. non-clinical), moderation analyses, using a mean-centered interaction term, were considered. However, as the bivariate associations for Machiavellianism and narcissism were non-significant, moderation was only tested for these traits using the PAQ NDAF subscale, which showed bivariate associations. For psychopathy, the overall model was significant [*R^2^* = 0.09, *F* (3, 421) = 13.81, *p* < 0.001] and a significant main effect of alexithymia was found (*B* = 0.018, *SE* = 0.0036, *t* = 4.93, *p* < 0.001), indicating that higher alexithymia was associated with higher psychopathy scores across both groups. However, the interaction term was not significant (*B* = −0.0035, *SE* = 0.0050, *t* = −0.70, *p* = 0.48). For Machiavellianism and narcissism, the moderation models using the PAQ NDAF subscale did not yield significant main or interaction effects (all *p* > 0.05).

## 4 Discussion

The present study assessed the Dark Triad, psychopathological symptoms such as depressive symptoms, anxiety, and stress and the use of emotion regulation strategies as well as alexithymia in a clinical and non-clinical population-based sample in Germany. In summary, the findings offer mixed support for the expected links between dark personality traits, alexithymia, emotion regulation, and psychological distress. Machiavellianism was consistently associated with higher anxiety and stress, underlining its role as a consistent correlate beyond other traits and emotion regulation processes. Narcissism was associated with stress, while psychopathy did not contribute independently once shared variance with other predictors was accounted for. Comparing the bivariate correlations with the regression results shows that some associations, such as the positive links between psychopathy and depressive symptoms or expressive suppression, did not hold when controlling for other variables. Similarly, the bivariate relationships between narcissism and psychopathological symptoms were mostly attenuated, remaining significant only for perceived stress. Notably, difficulties appraising positive affect (PDAF) explained additional variance in depression, anxiety, and stress, highlighting the relevance of positive affect processing for mental health. Group comparisons further indicated that dark personality traits did not differ significantly by treatment status, whereas levels of depression, anxiety, stress, and cognitive reappraisal varied as expected across clinical and non-clinical subgroups. These results emphasize the complex interplay between personality traits, emotion regulation, and alexithymia in understanding psychological distress.

Regarding psychopathy the expected relationship with psychopathological symptoms and emotion regulation was partially confirmed. There was a positive association between psychopathy and depressive symptoms, as well as between psychopathy and expressive suppression. These results align with previous findings, primarily based on non-clinical samples (Walker et al., 2022; Mojsa-Kaja et al., 2021; Bonfá-Araujo et al., 2023; Gómez-Leal et al., 2019; Burghart et al., 2024). Contrary to expectations, no significant negative association was found between psychopathy and cognitive reappraisal. Possible explanations may lie in the sample characteristics. Previous research that found a negative relationship between psychopathy and cognitive reappraisal did not include psychopathological symptoms (Walker et al., 2022). It is conceivable that this association may be attenuated or altered in a clinical sample, as psychopathological symptoms have themselves been linked to a reduced use of reappraisal (Aldao et al., 2010). This interpretation is further supported by the group comparisons conducted in this study, which showed that participants currently in treatment reported significantly lower use of reappraisal. Thus, the relationship between psychopathy and reappraisal could be influenced by clinical status; however, this potentially moderating effect was not explicitly tested in the present study and should be examined in future research. Importantly, when controlling for other dark traits, emotion regulation strategies, and alexithymia facets in the regression models, psychopathy did not show an independent association with depressive symptoms, anxiety, or stress. This suggests that its bivariate associations may largely reflect variance shared with Machiavellianism and other predictors.

As expected, positive associations were found between Machiavellianism and both anxiety and stress. These results are consistent with previous studies (Mojsa-Kaja et al., 2021; Lyons et al., 2019; Al Aïn et al., 2013; Birkás et al., 2020). However, there was no significant association with suppression. Previous research has reported mixed findings regarding this link (Walker et al., 2022; Akram and Stevenson, 2021). A recent meta-analysis did not find a relationship between Machiavellianism and either expressive suppression or cognitive reappraisal and the authors argued that the use of the Dirty Dozen, compared to other instruments, may produce weaker or even negative associations with emotion regulation strategies (Walker et al., 2022). Furthermore, the non-significant association between Machiavellianism and suppression in this study may be influenced by the heterogeneous distribution of diagnoses in the clinical sample. Prior research shows that the relationship between emotion regulation strategies and psychopathological symptoms can differ depending on the type of psychiatric disorder (Aldao et al., 2010). As the present study did not examine differences across specific diagnostic groups, these differences may have attenuated the overall effect. Machiavellianism remained significantly and independently associated with anxiety and perceived stress in the multivariate analyses, underlining its role as a robust association for psychological distress beyond other personality traits and emotion regulation processes.

For narcissism, positive associations were found with depression, stress, and anxiety. Previous research has reported mixed results for the link between narcissism and psychopathological symptoms, with grandiose narcissism often being associated with lower levels of psychopathology (Mojsa-Kaja et al., 2021; Bonfá-Araujo et al., 2023; Blasco-Belled et al., 2024). However, earlier studies indicate that grandiose narcissism can indirectly function as a buffer against psychopathological symptoms through resilience factors such as mental toughness (Papageorgiou et al., 2023). As this mediator was not assessed in the present study, this protective effect could not be examined. Moreover, the clinical nature of the sample may imply generally lower levels of resilience, which could weaken the self-esteem buffering function of grandiose narcissism. No significant associations were found between narcissism and the emotion regulation strategies assessed. This result is in line with previous studies that also did not find a link between grandiose narcissism and either expressive suppression or cognitive reappraisal (Walker et al., 2022). Past research has shown that vulnerable narcissism may be positively associated with suppression (Walker et al., 2022). Since the Naughty Nine primarily measures the grandiose core of narcissism, this finding is consistent with prior work (Walker et al., 2022). Narcissism remained independently associated with perceived stress in the regression models, while its associations with depression and anxiety did not hold when controlling for other traits and emotion regulation, highlighting its more limited contribution.

Regarding emotion regulation, cognitive reappraisal was consistently linked to lower levels of depressive symptoms and perceived stress, highlighting its potential buffering effect in emotion regulation, which should be confirmed in future longitudinal studies. Interestingly, no significant link was found for anxiety, suggesting that reappraisal may be less effective in reducing anxiety symptoms or that other strategies might be more relevant. This is further supported by the group comparisons, which indicated lower levels of reappraisal among participants currently in treatment, underlining its clinical relevance.

In addition to the main analyses, potential group differences and more differentiated associations between alexithymia and dark personality traits were explored. The results indicate that dark personality traits did not significantly differ between adults with and without treatment, suggesting that these traits may represent relatively stable risk factors relevant in both clinical and non-clinical contexts. Consistent with the results, the moderation analyses indicated that the positive association between alexithymia and psychopathy did not differ significantly between clinical and non-clinical participants, suggesting a stable link across groups. For Machiavellianism and narcissism, no significant moderation effect emerged for their association with difficulties in appraising negative emotions, in line with the weaker bivariate associations observed for these traits. Future research might explore whether different facets of alexithymia or more differentiated measures for Machiavellianism and narcissism show stronger links. The exploratory analysis of the associations between the selected PAQ subscales and the dimensions of the Dark Triad revealed that psychopathic traits were consistently positively associated with both alexithymia dimensions reflecting difficulties in appraising negative and positive emotions as well as the dimension reflecting externally oriented thinking and the total score. These consistent associations support the notion that psychopathic traits are closely linked to broad deficits in emotion recognition and appraisal, as highlighted in earlier studies (Jonason and Krause, 2013; Burghart and Mier, 2022; Di Tella et al., 2024). In contrast, the absence of robust correlations between Machiavellianism and alexithymia in the present study appears to diverge from some prior findings reporting significant links (Jonason and Krause, 2013; Di Tella et al., 2024; Al Aïn et al., 2013). This could be attributable to the focus on manipulative exploitation as the core dimension measured by the Naughty Nine, whereas other facets of Machiavellianism might relate differently to alexithymia (Christie and Geis, 1970; Küfner et al., 2015). Moreover, the generally higher levels of alexithymia in the clinical subgroup may have restricted variance, potentially attenuating the observed correlations with Machiavellianism. A similar explanation may apply to narcissism, as the Naughty Nine primarily captures the grandiose dimension of narcissism. Indeed, findings on the association between narcissism and alexithymia have been mixed overall, with studies reporting varying relationships, particularly for grandiose narcissism, and suggesting that these associations may differ depending on the specific facets of alexithymia assessed (Jonason and Krause, 2013; Di Tella et al., 2024). Overall, this pattern may suggest that the affective detachment typical of psychopathy inherently overlaps with difficulties in emotion awareness and appraisal, while the more strategic or self-enhancing elements of Machiavellianism and narcissism may not entail such broad deficits. Moreover, the regression results showed that the PDAF facet of alexithymia accounted for additional variance in depression, anxiety, and stress, even beyond the effects of dark personality traits and emotion regulation. This finding underscores the unique contribution of positive affect processing difficulties to psychological distress. Finally, in line with the Attention-Appraisal Model and previous evidence suggesting that alexithymia represents a transdiagnostic risk factor, the present findings confirmed significant positive associations between alexithymia and depressive symptoms, anxiety, and perceived stress (Weissman et al., 2020; Preece and Gross, 2023).

These results highlight that dark personality traits, emotion regulation, and alexithymia facets interact in complex ways to explain psychological distress, with Machiavellianism and difficulties appraising positive affect emerging as particularly robust factors. This underscores the importance of addressing both maladaptive traits and emotion regulation skills in clinical interventions. Moreover, the findings emphasize the need to consider the overlap between dark personality traits in interpretation and emphasize the importance of using more differentiated measures. Future studies should therefore aim to assess distinct facets, especially for narcissism and psychopathy, to better identify potentially opposing effects.

### 4.1 Strengths and limitations

The present study is subject to several limitations, which should be acknowledged. First, the cross-sectional design precludes any conclusions about causality. Although associations between variables were identified, it remains unclear whether these reflect directional or reciprocal relationships. However, the study’s exploratory approach provides first insights into these relationships and highlights potential directions for future longitudinal research. Second, the sample characteristics may limit the generalizability of the findings. The majority of participants were young, female, and highly educated, which may have introduced a sampling bias. Future studies should aim to include more diverse populations in terms of age, gender, and educational background. By including participants from both clinical and non-clinical contexts, the study aimed to enhance sample diversity and generalizability. At the same time, this design limits the extent to which conclusions can be drawn about one specific group alone. Furthermore, future studies that include clinical samples could benefit from examining differences across specific diagnostic groups, especially regarding emotion regulation. Third, as with all self-report measures, responses may have been influenced by social desirability or limited self-insight. Particularly in the assessment of socially aversive traits, underreporting is possible. However, it is possible that the anonymous online format may have helped to reduce social desirability bias to some extent by increasing perceived privacy. In addition, the inclusion of attentiveness checks helped to ensure data quality despite the online format. Fourth, some limitations pertain to the instruments used. As there are limited measurement instruments for the Dark Tetrad in German-speaking countries, the present study focuses solely on the Dark Triad. The individual subscales of the Dark Triad were measured using the Naughty Nine questionnaire, which serves to economically capture the core aspects of the Dark Triad. However, it does not allow for the assessment of all relevant subdimensions but rather focuses on capturing the core features of each construct (Küfner et al., 2015). This is a notable limitation, particularly regarding the measurement of narcissism, as different findings emerge when considering vulnerable and grandiose narcissism and the Naughty Nine primarily capture the grandiose core of narcissism (Küfner et al., 2015). Similarly, psychopathy was measured as a global construct, without differentiating between primary and secondary psychopathy, which could also affect the observed relationships. Despite these limitations, the Naughty Nine questionnaire provides a practical and economical German-language tool to explore initial associations between dark personality traits and other psychological constructs. Finally, the German version of the PAQ was used, which has been psychometrically validated in English but not yet in German. Its use enabled a more nuanced examination of alexithymia facets, adding depth to the analyses. Although it has been successfully applied in other German studies (Baumann et al., 2025), the results should nevertheless be interpreted with caution.

Despite the limitations discussed above, the study also offers several noteworthy strengths. The sample size and its composition enabled a range of statistical analyses and increased the power to detect meaningful associations. Moreover, the inclusion of a clinical subsample contributes valuable insights to a research field that has so far been only scarcely examined the German-speaking context, particularly regarding dark personality traits and alexithymia. In addition, the study provides a multifactorial perspective by including both alexithymia and emotion regulation, thereby identifying diverse theoretical and empirical implications for future research.

## 5 Conclusion

In summary, the present study provides insights into the relationships between dark personality traits, psychopathological symptoms, emotion regulation, and alexithymia in a clinical and non-clinical German sample. While Machiavellianism consistently emerged as a consistent correlate for anxiety and stress, bivariate results also highlight relevant links for psychopathy and narcissism, underscoring the need for more differentiated assessment. Moreover, the findings highlight a potentially protective role of cognitive reappraisal for depression and stress and underline the contribution of difficulties in appraising positive emotions to psychological distress. These results suggest potential research directions, for example, exploring whether anxiety-management strategies for individuals high in Machiavellianism, stress-reduction approaches for those high in narcissism, or positive-affect training aimed at alexithymia could be beneficial. Overall, this study strengthens the importance of addressing both maladaptive personality traits and emotion-processing deficits in prevention and intervention efforts. Future research should focus on distinguishing specific facets of the Dark Triad traits and further examine how emotion regulation and alexithymia interact with these traits, especially in clinical contexts.

## Data Availability

The raw data supporting the conclusions of this article will be made available by the authors, without undue reservation.
